# CAR-T cell therapy in T-cell malignancies: Is success a low-hanging fruit?

**DOI:** 10.1186/s13287-021-02595-0

**Published:** 2021-10-07

**Authors:** Pouya Safarzadeh Kozani, Pooria Safarzadeh Kozani, Fatemeh Rahbarizadeh

**Affiliations:** 1 Department of Medical Biotechnology, Faculty of Paramedicine, Guilan University of Medical Sciences, Rasht, Iran; 2 Student Research Committee, Medical Biotechnology Research Center, School of Nursing, Midwifery, and Paramedicine, Guilan University of Medical Sciences, Rasht, Iran; 3 Department of Medical Biotechnology, Faculty of Medical Sciences, Tarbiat Modares University, Tehran, P.O. Box 14115-111, Iran; 4 Research and Development Center of Biotechnology, Tarbiat Modares University, Tehran, Iran

**Keywords:** Chimeric antigen receptor, T-cell malignancies, Fratricide, T-cell aplasia, Natural killer cells, Cancer immunotherapy

## Abstract

Chimeric antigen receptor T-cell (CAR-T) therapy has been prosperous in the treatment of patients with various types of relapsed/refractory (R/R) B-cell malignancies including diffuse large B-cell lymphoma (DLBCL), B-cell acute lymphoblastic leukemia (B-ALL), follicular lymphoma (FL), mantle cell lymphoma (MCL), and multiple myeloma (MM). However, this type of therapy has faced serious hindrances in combating T-cell neoplasms. R/R T-cell malignancies are generally associated with poor clinical outcomes, and the available effective treatment approaches are very limited. CAR-T therapy of T-cell malignancies has unique impediments in comparison with that of B-cell malignancies. Fratricide, T-cell aplasia, and product contamination with malignant T cells when producing autologous CAR-Ts are the most important challenges of CAR-T therapy in T-cell malignancies necessitating in-depth investigations. Herein, we highlight the preclinical and clinical efforts made for addressing these drawbacks and also review additional potent stratagems that could improve CAR-T therapy in T-cell malignancies.

## Introduction

Cancer immunotherapy has helped patients with hematologic malignancies achieve durable clinical responses. Among various platforms of cancer immunotherapy, chimeric antigen receptor T-cell (CAR-T) therapy has been successful in the clinic in the case of relapsed/refractory (R/R) hematologic malignancies. *Tisagenlecleucel*, *axicabtagene ciloleucel*, and *lisocabtagene maraleucel* are three US Food and Drug Administration (FDA)-approved CAR-T products for the treatment of R/R diffuse large B-cell lymphoma (DLBCL) [1, 2]. All of these CAR-T products target CD19 as the target antigen. Moreover, *tisagenlecleucel* and *axicabtagene ciloleucel* have also been approved by the FDA for the treatment of B-cell acute lymphoblastic leukemia (B-ALL) and follicular lymphoma (FL), respectively [3]. Additionally, *brexucabtagene autoleucel* is another CD19-redirected CAR-T product that has received FDA approval for medical use in patients with mantle cell lymphoma (MCL) [4]. In addition to the mentioned products, *idecabtagene vicleucel* is the latest CAR-T product targeting B-cell maturation antigen (BCMA) and it has been approved by the FDA for the treatment of certain patients with multiple myeloma (MM) [5]. Despite all this clinical success, CAR-T therapy can only mediate limited responses in some types of hematologic malignancies and solid tumors [6]. T-cell malignancies are one of the fields in which CAR-T therapy has not yielded clinically favorable outcomes.

T-cell malignancies are an extensively heterogeneous group of diseases that are generally associated with discouraging prognosis [7]. T-cell neoplasms include malignancies that arise from T-cell precursors (for instance, thymocytes) such as T-cell acute lymphoblastic leukemia (T-ALL) and lymphoma and malignancies that arise from mature T cells such as T-cell large granular lymphocyte (LGL) leukemia, adult T-cell leukemia/lymphoma (ATL or ATLL), T-cell prolymphocytic leukemia (T-PLL), and a large number of peripheral T-cell lymphomas (PTCLs) [7]. As compared to B-cell malignancies, the use of first-line cancer treatment modalities including chemotherapy for patients with T-cell malignancies only helps achieve limited clinical responses resulting in the poor prognosis of such patients [8, 9]. So far, various types of immunotherapeutics have been developed for improving the clinical outcomes of patients with T-cell malignancies. Considering the pronounced clinical success of CAR-T therapy in B-cell malignancies, there is great interest for mediating successful outcomes in T-cell malignancies as well.

Regardless of this significant interest, CAR-T therapy of T-cell neoplasms has been rather challenging. The first challenge is the lack of T-cell neoplasm-specific CAR-T target antigens. Most of the antigens targeted in CAR-T therapy of T-cell neoplasms (such as CD3, CD5, and CD7) are expressed by normal T cells as well [10–18]. This phenomenon leads to the CAR-T-mediated eradication of normal T cells which is known as T-cell aplasia [19, 20]. T-cell aplasia can be life-threatening if not managed or prevented [19, 20]. In addition, the expression of the CAR-T target antigen by the CAR-Ts themselves leads to a phenomenon known as fratricide in which CAR-Ts attack and eliminate each other [21]. This incidence results in impaired CAR-T persistence and antitumor effects [21]. Alongside the well-known CAR-T-targeted T-cell neoplasm antigens, there are also other target antigens whose targeting might be beneficial in T-cell malignancies, even if with case-to-case variability [22, 23]. For instance, the aberrant expression of myeloid markers such as CD13 and CD33 has been detected in precursor T-cell leukemias which may presage a poor prognosis in comparison with T-cell leukemia cases without the expression of myeloid antigens [22, 23]. Such alternative target antigens might also be considered as immunotherapy targets alongside the conventional T-cell malignancy target antigens for achieving improved clinical responses. Above all these limitations, the generation of autologous CAR-Ts from the peripheral blood mononuclear cells (PBMC) of patients with T-cell neoplasms is very problematic since malignant and normal T cells are isolated together in the process of leukapheresis. In this case, the autologous CAR-T product might contain engineered T cells generated from malignant T lymphocytes. All of these limitations have significantly obstructed the way of successful CAR-T therapy in T-cell malignancies. In this review, we discuss the most potent preclinical and clinical studies that have tried to address these unique limitations of CAR-T therapy in T-cell malignancies. We also shine a light on the potential innovative strategies that can maximize the success and applicability of CAR-T therapy in the treatment of T-cell neoplasms.

## Fundamentals of CAR-T therapy and its limitations in T-cell malignancies

**Fig. 1:**
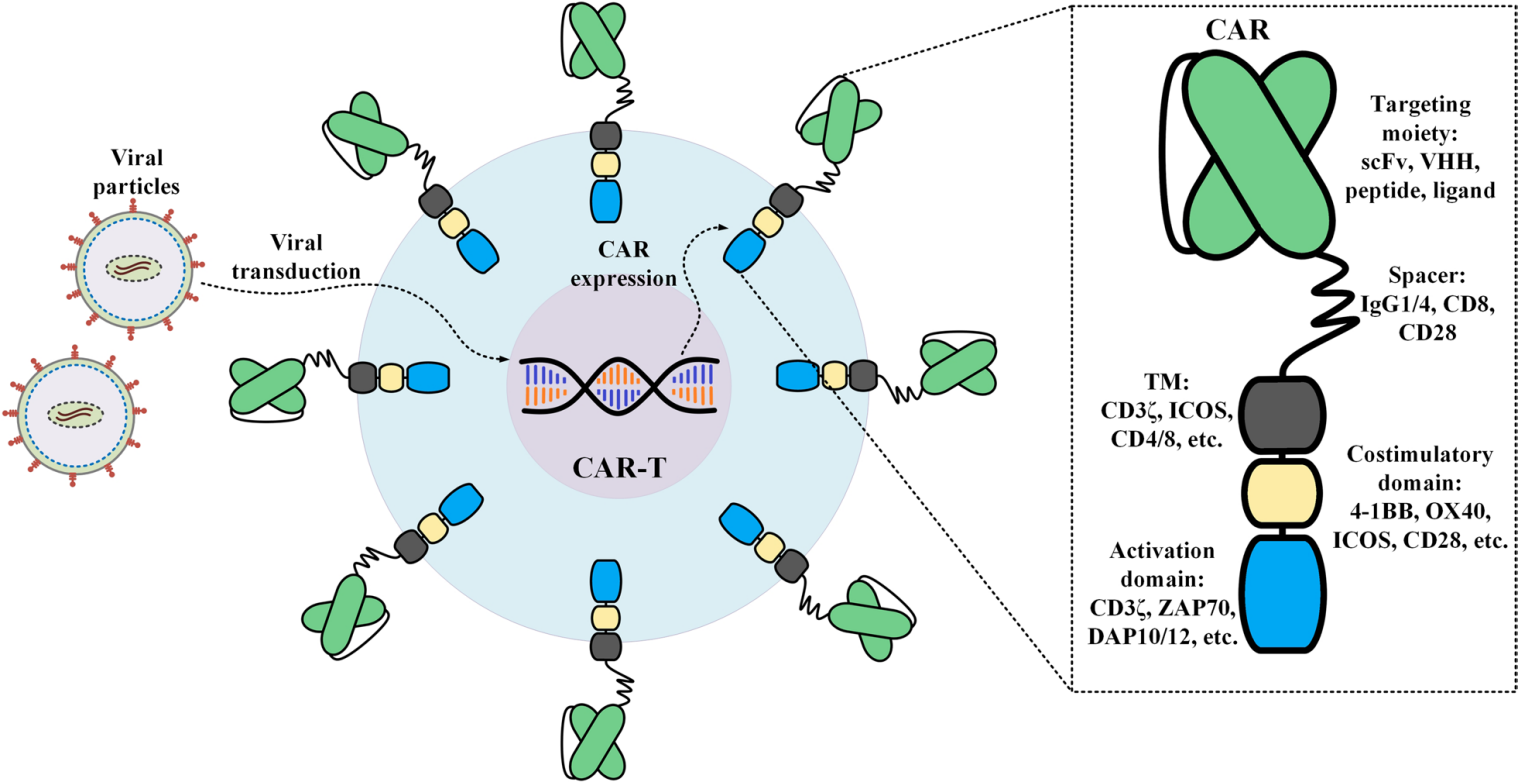
A detailed representation of a CAR molecule. Isolated T cells are transduced with viral particles and the gene fragment encoding the CAR constructs are integrated within their genome (as is in the case of CAR-T products approved for medical use in the US). This leads to the expression of the CAR molecules on the surface of the transduced T cells. In detail, a conventional CAR is composed of a targeting moiety followed by a spacer fragment, also known as a hinge, a transmembrane domain, a costimulatory domain, and an activation domain. So far, five generations of CARs have been devised by different investigational teams with different characteristics and components which have been comprehensively discussed elsewhere [36, 37]. *CAR* chimeric antigen receptor; *ICOS* the inducible T-cell co-stimulator; *scFv* single-chain variable fragment; *TM* transmembrane domain

CAR-T therapy is the result of years of development in various fields of basic and clinical studies. CARs are synthetic surface-expressed molecules that can grant T cells, or any other effector cells such as natural killer (NK) cells, the ability to concentrate their cytotoxicity on a specific type of target tumor cell expressing the CAR target antigen. CAR transgenes are either transiently or stably introduced into T cells [24, 25]. In detail, CAR mRNA electroporation results in transient CAR expression while lentiviral or gammaretroviral gene delivery approaches result in CAR transgene integration into the genome of T cells and its stable expression [24, 25]. The target antigens of CARs may either be tumor-specific antigens (TSAs) or tumor-associated antigens (TAAs) [26]. The ability of CARs for recognition and interaction with such target antigens is mainly dependent on their extracellular domain which is made of a targeting domain and a hinge (also known as “spacer”) [27]. The single-chain variable fragment (scFv) of a monoclonal antibody (mAb) is mostly used as the targeting domain of CARs [28]. However, nanobodies (also known as VHH) or toxins have also been used for the same purpose [29–35]. The hinge is the link between the extracellular domain and the transmembrane domain of CARs [36]. CARs also harbor an intracellular domain which includes an activation domain, generally CD3ζ derived from the CD3 complex of T-cell receptor (TCR), and one or two co-stimulatory domains including CD28, 4-1BB (CD137), ICOS, or OX40 (CD134) [36]. The extracellular domain and intracellular domain of CARs are connected by the transmembrane domain which also acts as an anchor for keeping the CAR molecules in place in the membrane (Fig. 1). When CAR molecules encounter their target antigen, the triggered downstream signaling pathway leads to T-cell activation. This intelligent mechanism of action does not involve major histocompatibility complex (MHC) for activation [36]. This ability of CARs enables the recognition of target antigens that have not been processed and presented by MHC molecules on antigen-presenting cells. Therefore, any surface-expressed antigen whose expression is mostly restricted to malignant cells, rather than normal ones, and can also be targeted using mAbs might be considered as a suitable CAR-T therapy target antigen [26, 27].

Currently, CAR-Ts are classified into five generations based on their intracellular domains. The first-generation CARs do not possess any co-stimulatory domains in their intracellular domain [37, 38]. These CAR-Ts demonstrated impaired and weak target antigen-dependent activation, expansion, and antitumor activity [36, 39–41]. These discouraging outcomes motivated researchers to engineer CAR-Ts capable of tackling the limitations of first-generation CAR-Ts [37, 38]. For this aim, researchers incorporated co-stimulatory domains into the intracellular domain of CARs and generated second- and third-generation CARs [36, 39–41]. The second-generation and the third-generation CARs have one and two co-stimulatory domains in their intracellular domain, respectively [37, 38]. These generations of CAR-Ts demonstrated enhanced target antigen-triggered activation, expansion, persistence, and antitumor activity in comparison with those of first-generation CAR-Ts [36, 39–41]. The fourth generation of CARs, which are also known as “T cells redirected for antigen-unrestricted cytokine-initiated killing” or “TRUCK”, are second-generation CAR-based receptors that harbor a cytokine expression inducer [37, 38]. This generation of CAR-Ts may appear beneficial in the case of solid tumors in which CAR-Ts sometimes are unable to attack malignant cells due to the antigen loss of the target cells [37, 38]. Fourth-generation CAR-Ts can produce and secret a particular cytokine, such as IL-12, which can be used to draw endogenous immune cells to attack the desired malignant cells [37, 38]. The fifth generation of CARs is also much similar to the fourth-generation but with the only difference that, instead of a cytokine expression inducer, they have an intracellular domain of a cytokine receptor [37, 38, 42]. These CAR-Ts can mediate target antigen-dependent activation of the JAK-STAT pathway that results in their enhanced expansion and persistence and prevents terminal differentiation [42].

**Fig. 2:**
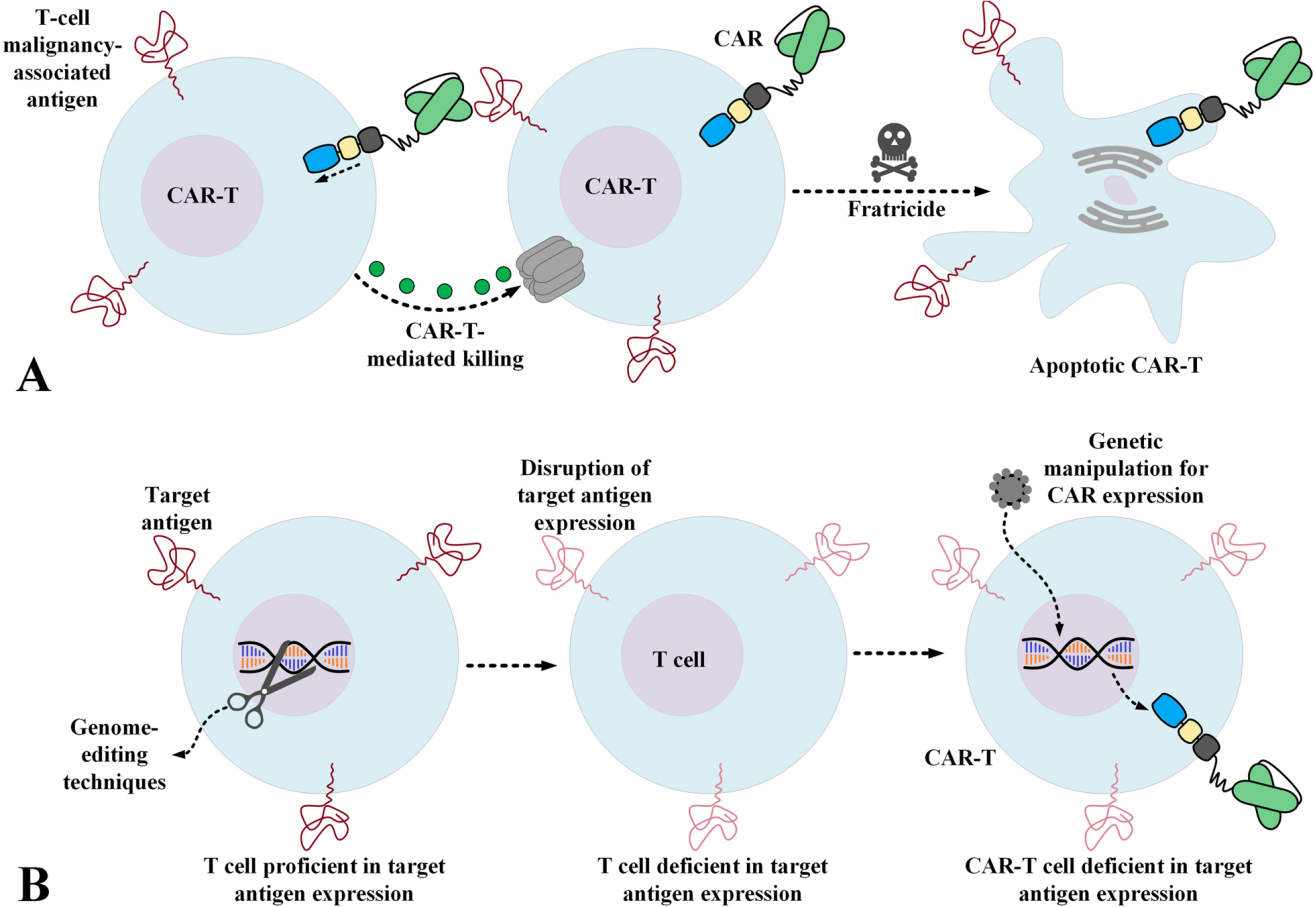
CAR-T-mediated fratricide (**A**) and how the disruption of the target antigen expression can prevent its occurrence (**B**)

CAR-T therapy of T-cell malignancies appears to be much more intricate than that of B-cell malignancies. In this case, a CAR-T redirected towards a TAA expressed by T cells can recognize normal T cells, malignant T cells, and also other CAR-Ts [21]. The expression of the CAR target antigen on the surface of CAR-Ts leads to a phenomenon called “fratricide”. Fratricide is the cytotoxicity of CAR-Ts against other CAR-Ts (Fig. 2A). This process leads to CAR-T-mediated eradication of CAR-Ts resulting in limited and poor in vivo expansion and impaired tumoricidal activity [21]. Additionally, most of the target antigens targeted by CAR-Ts in T-cell malignancies are also expressed by normal T cells [10, 13–16, 18, 21]. CAR-T-mediated eradication of normal T cells leads to an extremely substantial level of immunosuppression which can leave room for various types of life-threatening infections (which might lead to an elevated rate of mortality) [19, 20]. Moreover, in the case of malignancies other than T-cell neoplasms, for the aim of generating autologous CAR-Ts, T cells are isolated from the patient and are genetically engineered to express CARs. But in the case of T-cell malignancies, isolating only healthy non-malignant T cells is almost impossible and rather problematical since malignant T cells and normal T cells are isolated simultaneously from such patients. All of these limitations render CAR-T therapy of T-cell neoplasms problematic and challenging. In the upcoming sections, we will underscore studies that have tried to address these unique caveats paving the way for improved CAR-T therapy in T-cell malignancies.

## Target antigens, fratricide, and the proposed solutions

### Targeting Pan-T antigens

#### CD3

CD3 is a Pan-T surface antigen that forms a complex with the TCR enabling target antigen recognition and the subsequent signaling that leads to T-cell activation [18]. The exclusive membrane-associated expression and presence of CD3 on all mature T cells have rendered this antigen a favorable target for the immunotherapy of T-cell malignancies [18]. In 2009, Frankel et al. evaluated the anti-CD3 recombinant diphtheria immunotoxin in 5 patients with cutaneous T-cell lymphoma (CTCL) [17]. They reported a half-life of less than an hour and anti-immunotoxin neutralizing antibodies developed in all of the patients after two weeks [17]. According to the results of this study, 2 of the patients (40%) had partial disease remission, one of which lasted more than 6 months [17]. In 2015, Frankel et al. reported the results from a clinical trial (NCT00611208) evaluating an anti-CD3ε second-generation recombinant diphtheria immunotoxin in 30 patients (25, 3, 1, and 1 with CTCL, PTCL, T-cell LGL leukemia, and T-PLL, respectively) [43]. Among the patients with CTCL, only 4 (16%) achieved complete remission (CR) with durations varying from more than 36 months to more than 72 months [43]. However, since such responses are poor and unsatisfactory, they create an increasing need to develop more efficient approaches.

Using CD3 as a CAR-T therapy target in T-cell malignancies leads to poor responses due to fratricide. Therefore, generating fratricide-resistant CAR-Ts or using other types of effector cells such as NK-92 cells might somewhat eliminate the mentioned caveats [44, 45]. Genome editing methods can offer various routes for the disruption of an antigen of interest such as CD3. Researchers have utilized transcription activator-like effector nuclease (TALEN)-assisted disruption of the endogenous TCRαβ/CD3 of T cells before genetically manipulating these cells to express the CD3ε-targeting CARs (Fig. 2B) [45]. These CAR-Ts demonstrated specific and significant tumoricidal activity against primary T cells and childhood samples of T-ALL [45]. This specific cytotoxicity was also confirmed in preclinical models engrafted with the CD3^+^ Jurkat cell line [45]. Moreover, other researchers engineered third-generation CD3-targeting CARs and expressed them in the NK cell line NK-92 [44]. NK cells are deficient in CD3 expression and have shorter lifecycles as compared to those of T cells [44]. In the study by Chen and co-workers, the exclusive antitumor activity of these CAR-NKs was confirmed against CD3-expressing PTCL samples and various T-cell leukemia cell lines ex vivo [44]. Furthermore, these effector cells mediated effectively exclusive in vivo tumor outgrowth repression in preclinical models engrafted with the Jurkat cell line and extended their survival notably [44].

#### CD5

CD5 is a membrane-spanning glycoprotein that has an extracellular domain [46–48]. This antigen plays roles in the survival of human lymphocytes and also operates as a cellular component that negatively regulates TCR signaling [46–48]. The normal expression of CD5 is exclusively observed in thymocytes, peripheral T lymphocytes, and a subpopulation of B lymphocytes named B-1a cells [15, 16]. The aberrant expression of CD5 is detected in several T-cell malignancies including T-ALL and PTCL [10, 13, 14]. mAbs targeting CD5 and immunotoxins generated using CD5-specific mAbs have been investigated in patients with different types of T-cell malignancies including T-ALL and CTCL [49, 50]. The results of these investigations demonstrated high levels of specificity alongside low levels of unwanted adverse events suggesting the suitability of this antigen for the immunotherapy of different types of T-cell malignancies [49, 50].

In 2015, Mamonkin et al. generated CD5-redirected CAR-Ts and reported that these cells experienced partial and transient fratricide and were capable of mediating exclusive antitumor activity against T-ALL and T-cell lymphoma cell lines in vitro [51]. The researchers also investigated the exclusive tumoricidal capacity of these CAR-Ts in xenograft T-ALL preclinical models and reported that, despite the complete elimination of malignant cells in vitro, CD5-redirected CAR-Ts suppressed tumor expansion in animal models but failed to completely eradicate the tumor [51]. Therefore, it was concluded that the disease relapse did not result from antigen loss in these preclinical animal models [51]. In 2017, Chen and co-workers used NK cells for the expression of CD5-redirected CARs [52]. Since NKs do not express CD5 on their surface, fratricide is no longer considered a limitation in this case [52]. These researchers expressed third-generation CARs in the NK-92 cell line and demonstrated that, alongside steady expansion ex vivo, these CAR-NKs mediated exclusive and pronounced tumoricidal activity towards primary CD5^+^ cells of human T-ALL and PTCL and T cell lines including Jurkat, CCRF-CEM, and MOLT-4 [52]. Chen et al. also reported improved survival of mouse xenograft models along with substantial suppression of established T-ALL tumors [52]. Other studies have also reported that NK-92 cells exhibit exclusive antitumor responses against T-cell leukemia cell lines and established T-ALL in xenograft models [53]. In 2018, Raikar et al. utilized CRISPR-Cas9 to knockout CD5 in T cells (both Jurkat and primary cells) before transferring the CAR transgene into them [53]. They reported an elevated level of CAR expression along with a decreased level of fratricide resulted from self-recognition of CD5 by the CAR-Ts [53].

In 2018, Mamonkin et al. reported that using the 4-1BB co-stimulatory domain in the endodomain of CD5-redirected CAR-Ts, instead of CD28, can promote central memory differentiation in the CAR-Ts leading to enhanced tumoricidal activity [54]. However, they reported a boosted level of fratricide and a reduced level of expansion capacity of these CAR-Ts in comparison with those of CD5-redirected CAR-Ts harboring the CD28 co-stimulatory domain [54]. Additionally, these researchers investigated a Tet-Off system capable of interfering with CAR signaling and avoiding T-cell fratricide and target antigen exhaustion ex vivo by controlling the CAR surface expression in a reversible manner [54]. This system enables reversible repression of CAR expression in the presence of doxycycline ex vivo while CAR surface expression can be recovered in vivo when there is no doxycycline administration; therefore, central memory development can be maintained without the immediate onset of fratricide [54]. These researchers concluded that 4-1BB-based CD5-redirected CAR-Ts equipped with the Tet-Off system demonstrated superior and more prolonged leukemia repression in comparison with CD28-based CD5-redirected CAR-Ts in preclinical xenograft models [54]. In 2019, Xu and colleagues investigated the effects of using the 2B4 co-stimulatory domain in the construct of CARs expressed in the NK-92 cells in comparison with 4-1BB-based CARs [55]. These researchers used the Jurkat, MOLT-4, and MAVER-1 cell lines expressing CD5 for in vitro antitumor functionality assessments and confirmed the exclusive antitumor activity of CAR-NKs harboring either the 2B4 or 4-1BB co-stimulatory domain [55]. Moreover, they confirmed the target antigen specificity of these CAR-NKs against MV4-11 cells (which are deficient in the expression of CD5) [55]. In xenograft T-ALL preclinical models, both of the mentioned CAR-NKs demonstrated significant levels of tumoricidal activity with the CAR-NKs harboring the 2B4 co-stimulatory domain showing an enhanced antileukemic activity [55].

#### CD7

CD7 is a membrane-spanning glycoprotein from the Ig superfamily that is normally expressed on NK cells and T lymphocytes [12]. Studies have shown that a high percentage of T-ALLs and T-cell lymphomas are CD7-proficient [10, 11]. Moreover, immunotoxins generated using CD7-specific mAbs have also been studied in preclinical and clinical investigations against T-cell leukemias and lymphomas [56, 57].

Production of CAR-Ts targeting CD7 has been problematic since T cells themselves express CD7 which can result in fratricide and compromised expansion of the CAR-T product [11]. In 2017, Gomes-Silva and colleagues used the CRISPR-Cas9 genome editing tool to disrupt CD7 expression in T cells before engineering them for CAR expression [11]. They reported that not only did this approach not have any negative effects on the tumoricidal functionality of these CAR-Ts but it also resulted in their improved expansion efficiency [11]. These CAR-Ts demonstrated exclusive antitumor activity against various CD7-expressing cell lines, including Jurkat, CCRF, MOLT-4, Sup-T1, and Hut78, and primary cell samples from T-ALL patients with variable levels of CD7 expression [11]. In vivo assessments also demonstrated parallel results as these CAR-Ts exhibited strong tumoricidal activity against T-ALL in xenograft models [11].

Moreover, other researchers have proposed a strategy for the reduction of CD7 expression and fratricide prevention [58]. In detail, they have used a protein expression blocker (PEBL) system made of a CD7-targeting scFv fused to a retention domain which leads to the entrapment of the antigen in the ER/Golgi and prevents its normal expression [58]. These researchers used this system in T cells before second-generation CD7-redirected CAR expression and reported that this method mitigated fratricide and it did not have any negative impacts on the expansion, INF-γ and TNF-α production, and the antitumor activity of CD7-redirected CAR-Ts [58]. Furthermore, these CAR-Ts demonstrated robust antileukemic activity in in vitro cytotoxicity assays on CD7-proficient cell lines (including Jurkat, Loucy, MOLT-4, KG1a, and CCRF-CEM) and in vivo assessments in cell line-established and patient-derived xenografts (PDX) models of T-ALL [58].

Using CRISPR-Cas9, Cooper et al. generated CD7-redirected CAR-Ts deficient in the expression of CD7 and TCR alpha chain (TRAC) that demonstrated effective tumoricidal activity against T-ALL primary cell samples and cell lines and suppressed tumor progression in PDX preclinical models without graft-versus-host disease (GvHD) mediation [59]. These researchers concluded that such approaches might be useful for generating allogeneic CAR-Ts for the treatment of various types of T-cell malignancies [59].

NK cells have also been used for the generation of CD7-redirected CAR-equipped effector cells [60]. You et al. generated monovalent and bivalent CD7-redirected CAR-NKs using CD7-specific nanobodies and NK-92MI cells [60]. These researchers reported that both of these CAR-NKs demonstrated effective and exclusive antitumor activity towards cell lines of T-cell leukemia and primary tumor cell samples of patients [60]. However, they reported that the bivalent CD7-redirected CAR-NKs exhibited superior tumoricidal activity against primary T-ALL cells and secreted more granzyme B and IFN-γ in comparison with those of monovalent CD7-redirected CAR-NKs [60]. Additionally, similar results were also obtained in preclinical assessments as bivalent CD7-redirected CAR-NKs considerably suppressed tumor outgrowth in xenograft models of T-ALL [60].

In the first-in-human clinical trial (NCT04004637) of CD7-redirected CAR-Ts, researchers used CD7-specific nanobodies as the targeting domain of CAR-Ts and investigated these cells in patients with CD7^+^ R/R T-ALL/T-cell lymphoblastic lymphoma (T-LBL) [61]. Moreover, they used an intelligent technique for the prevention of CD7 surface expression and the ensuing fratricide by keeping the antigen in the ER and/or Golgi [61]. In detail, these CAR-Ts demonstrated robust expansion and acceptable persistence in 2 out of 3 patients (66%), and minimal residual disease (MRD)-negative CR was reported in these patients in just less than one month [61]. Furthermore, the circulating abnormal T cells became undetectable in 2 of these patients (66%) after CAR-T administration [61]. However, variable levels of cytokine release syndrome (CRS) were observed in all of the patients alongside elevated levels of IL-6 [61]. Ultimately, the researchers concluded that CD7-redirected CAR-Ts may have considerable immunotherapeutic value for the treatment of patients with T-cell malignancies [61]. Other clinical trials (NCT04033302 and NCT03690011) are also investigating the suitability and efficacy of this target antigen in various types of T-cell malignancies.

### Targeting antigens with restricted expression

#### CD1a

CD1a is cell surface antigen present on cortical T-ALL cells (which is a major subgroup of T-ALL) [62–64]. The specific expression of this antigen has also been observed in developing cortical thymocytes [65]. T cells and CD34^+^ progenitor hematopoietic cells do not demonstrate CD1a expression [65]. This characteristic of CD1a renders it a suitable target antigen whose targeting could minimize the possibility of on-target off-tumor toxicity occurrence [65]. One research group generated CD1a-targeting CAR-Ts and reported that, alongside being fratricide-resistant, these CAR-Ts demonstrated strong tumoricidal capacity against CD1a-expressing T-ALL cell lines and primary cells of cortical T-ALL samples [65]. Moreover, in vivo assessments in cell line-established and PDX preclinical models of cortical T-ALL showed that these effector cells exhibit prolonged persistence and considerable antitumor activity following administration [65]. Such data might underline the suitability of CD1a for CAR-T therapy of cortical T-ALL; however, careful clinical evaluations are still required for more substantiated conclusions.

#### CD4

For more than two decades, CD4-specific mAbs have been investigated in clinical trials for the treatment of T-cell lymphomas [66–69]. Findings have reported acceptable efficacy, low levels of immunogenicity, and satisfactory levels of tolerability for these mAbs in eradicating CD4^+^ T cells [66–69]. In 2016, Pinz et al. generated third-generation CD8^+^ CD4-redirected CAR-Ts and reported that these cells exhibited exclusive antitumor activity towards a CD4-expressing cell line (KARPAS 299 cells) and patient-derived PTCL cell samples while retaining their memory stem cell-like phenotype [70]. Additionally, these researchers established preclinical mouse models using KARPAS 299 cells and demonstrated that CD4-redirected CAR-Ts were capable of mediating antitumor responses and protecting mouse models from tumor progression, and extending their survival rate in comparison with GFP-expressing T cells (control) [70]. Moreover, in 2017, Pinz et al. generated third-generation CD4-redirected CAR-NKs using the NK-92 cell line [71]. In vitro assessments demonstrated that these CAR-NKs exclusively eradicated various types of CD4-proficient patient-derived cell samples and cell lines of T-cell lymphoma and leukemia (including CCRF-CEM, KARPAS-299, and HL60) in a dose-dependent fashion [71]. Moreover, these researchers established xenograft models using trackable luciferase-harboring KARPAS-299 cells and reported that their CD4-redirected CAR-NKs mediated efficient antitumor activity against the malignant cells and extended the survival of the preclinical xenograft models [71]. Such findings highlight the tumoricidal capacity of CD4-redirected CAR-NKs in eradicating the hard-to-reach lymphoma nodules [71].

However, some researchers have indicated that CAR-mediated CD4 targeting in T-cell malignancies may be considered as an impermanent and bridging approach since the eradication of normal CD4^+^ T cells results in T-cell aplasia and the ensuing HIV/AIDS-like syndrome [72]. In this regard, Ma et al. used *alemtuzumab*, which is a CD52-specific humanized mAb, as a natural safety switch to eradicate the administered CD4-redirected CAR-Ts to prevent T-cell aplasia in preclinical models [73]. Of note, both normal and malignant lymphocytes express CD52 on their surface [73]. These researchers reported > 95% decline in the number of circulating CD4-redirected CAR-Ts both 6 h and 48 h after the administration of *alemtuzumab* [73]. Ultimately, Ma and colleagues concluded that such data may back up the application of *alemtuzumab* for the rapid depletion of CAR-Ts from the circulation as an attempt to prevent unwanted toxicities [73]. However, the safety and efficacy of CD4-redirected CAR-Ts for the treatment of T-cell malignancies are yet to be determined in the future clinical trials, some of which have already started (NCT03829540).

#### CD30

CD30, also known as TNFRSF8, is expressed by T and B cells after they received the activation signal upon target antigen stimulation [74]. CD30 expression has been documented in various T-cell malignancies including T-ALL and anaplastic large cell lymphoma (ALCL) [74]. There is scientific evidence that high-dose chemotherapy mediates an increased CD30 expression in T-ALL patients [75]. Such data may point out the fact that CD30 targeting might be a potential option for R/R T-ALL cases [75].

*Brentuximab vedotin* is an antibody–drug conjugate (ADC) composed of a CD30-specific mAb conjugated to *monomethyl auristatin E* (MMAE), which is an anticancer agent capable of disrupting microtubules [76]. The FDA has approved the use of this ADC for the treatment of patients with relapsed Hodgkin lymphoma (HL), ALCL, classical Hodgkin lymphoma (cHL), and PTCL [76]. Such clinical success might highlight the suitability of CD30 as an immunotherapy target for the treatment of various subtypes of T-cell malignancies [76].

Preclinical studies investigating the suitability of CD30 in CAR-T-assisted targeting go back to at least 20 years ago [77, 78]. Today, CD30-redirected CAR-Ts are being investigated at different stages of clinical trials. In 2017, Ramos et al. published a report from a phase I dose-escalation clinical trial (NCT01316146) with 7 R/R HL or 2 ALCL patients who received CD30-redirected second-generation CAR-Ts [79]. The investigators reported no CAR-T-related toxicities during this study [79]. Moreover, of 7 HL patients in this study, 2 entered CR (with one lasting more than 2.5 years and the other lasting about 2 years) and 3 HL patients experienced transient stable disease [79]. Moreover, only one of the 2 ALCL patients experienced CR lasting 9 months [79]. In the same year, a report from another clinical trial (NCT02259556) involving 18 patients with progressive R/R HL stated that high-grade toxicities occurred in only 2 of the patients, and CAR-T infusion was well-tolerated in the rest of the patients [80]. In terms of efficacy, 7 patients experienced partial remission and 6 patients had stable disease [80].

Moreover, other clinical trials (NCT02690545 and NCT02917083) have aimed to investigate the impact of different conditioning regimens on the efficacy of CD30-redirected CAR-Ts since using no reconditioning therapy in patients receiving CD30-redirected CAR-Ts has led to limited and impaired in vivo CAR-T expansion [81, 82]. In these trials, patients with R/R HL received conditioning therapy with *bendamustine* alone, *bendamustine* and *fludarabine*, or *cyclophosphamide* and *fludarabine* [81, 82]. The findings of these trials by Ramos et al. indicated that out of 41 patients, only 10 developed CRS (grade 1 only) and no neurotoxicity was observed during these studies [82]. It was also concluded that CAR-T in vivo expansion was dose-dependent and that *fludarabine*-based preconditioning therapy resulted in durable responses with an acceptable safety profile [82].

#### CD37

CD37 is a tetraspanin leukocyte-exclusive surface antigen that is expressed on mature normal and transformed B cells [83, 84]. CD37 has also regulatory roles in T-cell proliferation [83]. CTCL and PTCL are among T-cell malignancies in which CD37 expression has been detected [85, 86]. The safety, pharmacokinetics, and antitumor functionality of CD37 targeting via an ADC named *AGS67E* has been studied in a phase I dose-escalation clinical trial (NCT02175433) [87]. *AGS67E* is a fully human CD37-specific mAb conjugated to MMAE [87]. According to the results of this trial, alongside a promising safety index, *AGS67E* was capable of mediating antitumor activity especially in CTCL patients [87].

In 2018, Scarfò et al. generated CD37-redirected CAR-Ts and reported that these cells mediated target antigen-dependent activation, cytokine secretion, and tumoricidal activity against T-cell lymphomas in vitro without any significant signs of fratricide [88]. The in vitro assessments of this study involved cell lines such as Hut78 and Fedp and patients-derived cell samples of PTCL, all with variable levels of CD37 expression [88]. This study also reported the target antigen-dependent antitumor activity of dual-specific CAR-Ts redirected against CD19 or CD37 upon encountering either of the target antigens [88]. However, these researchers proposed that since not all PTCL cell lines or patient-derived samples are CD37^+^, screening for the expression of this antigen may be required in future preclinical and clinical investigations [88].

#### CCR4

C–C chemokine receptor type 4 (CCR4) is a chemokine receptor expressed by normal T-cell subsets including regulatory T cells (Tregs), T_h_2, and T_h_17 cells [89–91]. Moreover, the overexpression of this chemokine receptor has been detected in the malignant T cells of patients with ATLL, PTCL, and CTCL including *mycosis fungoides* (MF) and *Sézary syndrome* (SS) [89–92]. *Mogamulizumab* is a humanized mAb capable of targeting CCR4 [93]. This mAb has been approved by the FDA for the treatment of adult patients with R/R MF or SS who have not been responsive to at least one previous treatment approach [93]. *Mogamulizumab* is known as a first-in-class inhibitor for CCR4 and it has been available as a treatment for patients with R/R CTCLs [93].

In 2017, Perera et al. demonstrated that CCR4-redirected CAR-Ts generated from donor-derived T cells mediated effective target antigen-dependent antitumor responses against CCR4-expressing patient-derived tumor cell lines [94]. Moreover, these researchers also added that these CAR-Ts exhibited tumoricidal responses in a xenograft model of adult T-cell leukemia, which might further highlight the possible suitability of this antigen for the treatment of T-cell malignancies [94]. However, the expression of CCR4 on normal T-cell subsets might lead to unexpected toxicities which may require further in-depth assessments. Such unexpected toxicities, which include skin-related disorders (such as *Stevens-Johnson syndrome*), have been reported in patients treated with *mogamulizumab* [95–97].

#### TRBC1 and TRBC2

Malignant T cells exhibit significant down-regulation of TCR [98, 99]. However, TCR is expressed by almost one-third of T-ALLs and a great proportion of lymphoma cells [98, 99]. Most of PTCLs are TCR^+^, as some studies suggest that TCR signaling is required for their survival [100, 101]. In detail, TCRs possess an alpha chain and a beta chain. Either the T-cell receptor beta constant 1 (TRBC1) gene or the T-cell receptor beta constant 2 (TRBC2) gene is responsible for the expression of the TCR beta chain constant region [102]. Therefore, in a normal population of T cells, there will be a mixture of the cells expressing TRBC1 and cells expressing TRBC2 [103]. On the contrary, the whole population of malignant T cells will express either only TRBC1 or TRBC2 [103]. In this regard, researchers have proposed targeting TRBC1 (in the case of TRBC1-expressing T-cell malignancies) or targeting TRBC2 (in the case of TRBC2-expressing T-cell malignancies) [103]. The intelligence of this approach lies behind the fact that this method can eliminate cancerous T cells and a proportion of normal T cells expressing the target beta chain constant region but does not mediate any tumoricidal effects against a significant proportion of normal T cells [103].

Maciocia and colleagues generated TRBC1-redirected CAR-Ts and reported that these cells mediated exclusive antitumor responses against malignant TRBC1^+^, and not TRBC2^+^ cells, in vitro [103]. These researchers also stated that these CAR-Ts eradicated normal TRBC1^+^, and not TRBC2^+^ cells, in a specific fashion [103]. Moreover, NOD-SCID gamma (NSG) mice injected with TRBC1-TCR Jurkat T cells were treated with TRBC1-redirected CAR-Ts and irrelevant control CAR-Ts [103]. In detail, mice treated with TRBC1-redirected CAR-Ts demonstrated a significant tumor burden reduction and extended survival time as compared with the control group [103].

Stepping further, disrupting the expression of one of these TRBC genes using genetic engineering approaches results in the absence of the endogenous TCR from the cell surface [104]. This method can be used for the prevention of fratricide when generating autologous TRBC-redirected CAR-Ts [104]. In a nutshell, it may also be concluded that this strategy can be applied for the prevention of T-cell aplasia in the CAR-T therapy of T-cell malignancies; however, further clinical investigations are still required for more substantiated conclusions. An ongoing clinical trial (NCT03590574) is currently investigating the safety and efficacy of a TRBC1-targeting CAR-T therapy named *AUTO4* in patients with TRBC1^+^ T-cell non-Hodgkin lymphoma (T-NHL), PTCL, angioimmunoblastic T-cell lymphoma (AITL), and ALCL.

## T-cell aplasia and the proposed solutions

T-cell aplasia is the result of on-target off-tumor targeting of CAR-Ts against normal T cells expressing the CAR-specific target antigen. T-cell aplasia significantly elevates the risk of various kinds of life-threatening infections [19, 20]. Therefore, preventing this adverse event is highly required for successful CAR-T therapy in patients with T-cell malignancies. The occurrence of T-cell aplasia may be prevented through a variety of proposed strategies. One strategy is the selection of the target antigen. Targeting an antigen that is absent on normal T cells or it has an expression on a proportion of normal T cells might leave at least a percentage of normal T cells intact during CAR-T therapy. Another strategy is using CAR-Ts with limited or controllable life-span or activity whose limited antitumor effects can be beneficial in the prevention of T-cell aplasia onset. Moreover, bridging to allogeneic hematopoietic stem cell transplantation (HSCT) after CAR-T therapy might also be another option for mitigating CAR-T-associated T-cell aplasia [19].

The mutual expression of a target antigen between malignant and normal T cells leads to off-tumor toxicities in CAR-T therapy [105]. While targeting some target antigens might lead to the eradication of most of the T cells causing T-cell aplasia, targeting others might result in on-target off-tumor toxicities only against a subset of T cells. Therefore, the intact subsets of normal T cells during CAR-T therapy might sufficiently confer immunity. As discussed earlier, the strategy proposed by Maciocia and co-investigators uses TRBC1 (when the T-cell malignancy is TRBC1^+^) or TRBC2 (when the T-cell malignancy is TRBC2^+^) as CAR-T targets [103]. Targeting one of the mentioned targets in a patient does not lead to CAR-T-mediated eradication of the normal T cells expressing the other TCR beta chain constant region [103]. Moreover, it is worth mentioning that the target antigens discussed in the previous section have case-to-case variability even in a single type of T-cell malignancy. Therefore, choosing the right target antigen and considering the possibility of T-cell aplasia occurrence is a remaining subject of CAR-T therapy in T-cell neoplasms.

**Fig. 3:**
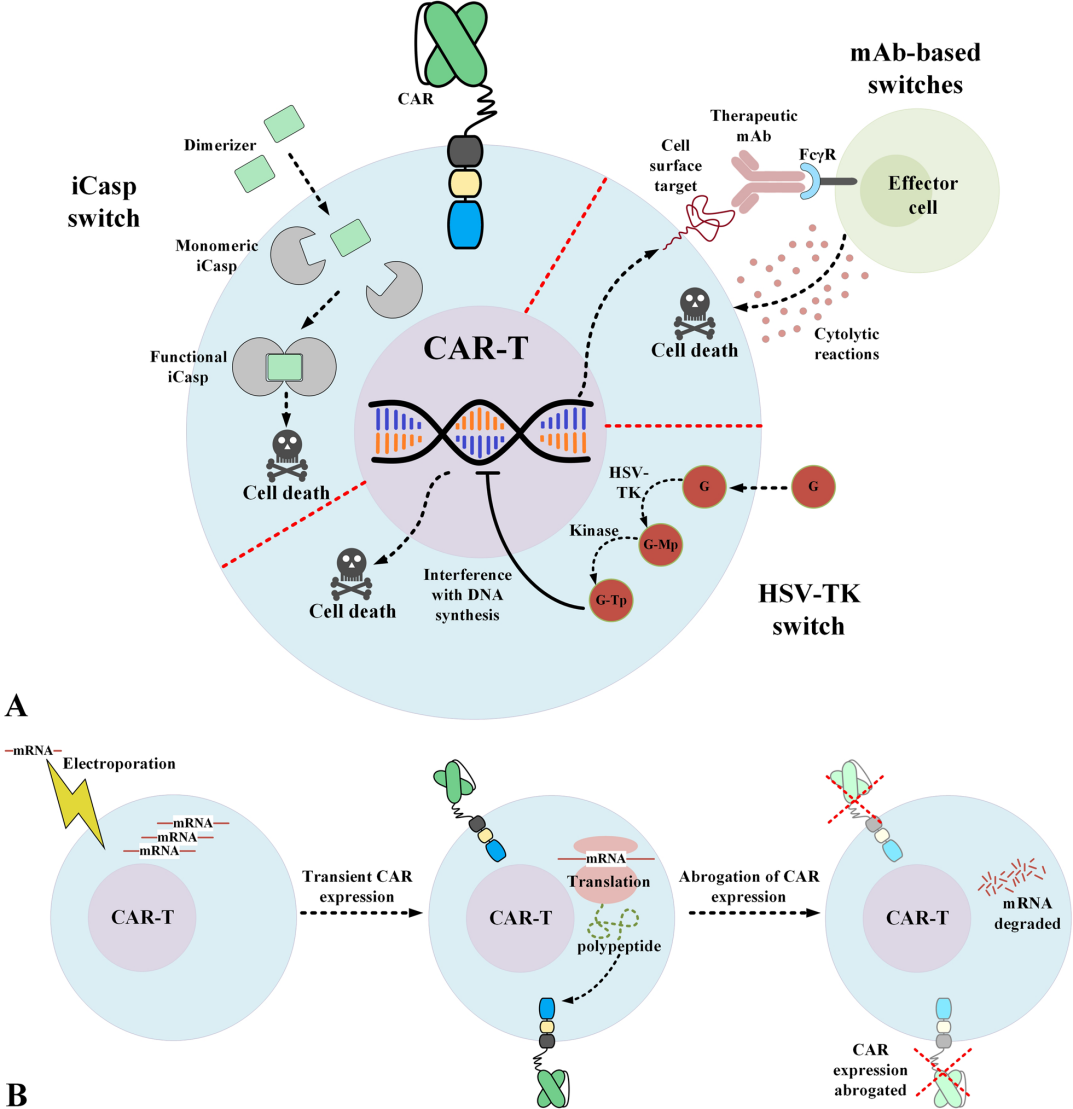
Potential strategies for overcoming the issue of prolonged CAR-T persistence which leads to T-cell aplasia. **A** Selective elimination of the administered CAR-Ts that is achievable through equipping the CAR-Ts with different safety switches. **B** Transient CAR expression that can be achieved through the mRNA electroporation of the isolated T cells. *CAR* chimeric antigen receptor; *FcγR* Fcγ receptor; *G* ganciclovir; *G-Mp* ganciclovir-monophosphate; *G-Tp* ganciclovir-triphosphate; *HSV-TK* Herpes simplex virus thymidine kinase; *iCasp* inducible caspase; *mAb* monoclonal antibody

Another applicable strategy for preventing T-cell aplasia is equipping CAR-Ts with safety switches (also known as suicide switches) which enable controlling the adoptively transferred T cells after their administration into patients (Fig. 3A) [36, 106]. So far, different platforms of safety switches have been introduced which include metabolic switches, mAb-dependent switches, and inducible caspase (iCasp) switches [36]. Each of these switches has advantages and disadvantages overanother and, depending on the need and situation, they might be applicable [36]. For instance, iCasp switches are based on the human caspase gene; therefore, the risk of immunogenicity is ruled out in this case [36, 106, 107]. On the other hand, metabolic switches, such as those based on herpes simplex virus thymidine kinase (HSV-TK), can be intertwined with immunogenicity issues due to their viral origin [108]. Also, in the case of mAb-based switches, the selective elimination of the adoptively transferred T cells is achieved upon the systemic administration of the specific mAb whose target antigens is engineered to be expressed by CAR-Ts [26, 36]. Since healthy tissues might also express this particular target antigen, they might be susceptible to being targeted by these mAbs, which might result in unwanted adverse events [26, 36]. Despite these notes, multiple research teams are currently investigating the applicability of such safety switches in clinical settings (NCT02028455, NCT03016377, or NCT01815749). The outcomes of these trials will likely elucidate the applicability of such safety switches and, if successful, they can be implemented for controlling the presence of CAR-Ts in the circulation after the onset of the early signs of T-cell aplasia or after the completion of the treatment to prevent T-cell aplasia.

Generating CAR-Ts using viral vector transduction results in the stable expression of CAR in the transduced T cells [109]. These transduced T cells can expand in vivo while maintaining CAR expression [109]. This open-ended duration of CAR expression leads to on-target off-tumor toxicities such as T-cell aplasia. CAR-Ts generated using CAR mRNA electroporated into T cells have demonstrated restricted and limited persistence after administration (Fig. 3B) [109–111]. Svoboda et al. have reported results from two pilot clinical trials (NCT02277522 and NCT02624258) investigating the use of non-viral mRNA-electroporated CD19-redirected CAR-Ts in patients with R/R HL [109]. According to this report, transient responses and no signs of severe toxicities were observed during these trials [109]. Considering the fact that this method might be beneficial in reducing the on-target off-tumor toxicities of CAR-T therapy in patients with T-cell malignancies, it is still speculated that sequential CAR-T administrations may be required for steady and reliable antitumor responses [27]. Also, it is worth mentioning that there are significant similarities between tumoricidal responses induced by non-viral mRNA CAR-Ts and virally transduced CAR-Ts [112].

## CAR-T product contamination with malignant T cells and the proposed solutions

Generating autologous CAR-Ts from patients with T-cell malignancies is challenging since during the process of T-cell isolation, both normal and malignant T cells are isolated. Therefore, after the CAR transgene introduction process, the CAR-T product population will contain malignant T cells as well as normal ones. In 2018, Ruella et al. reported that during the generation process of CD19-redirected CAR-Ts, one leukemic B cell was unintentionally genetically manipulated to express the CD19-redirected CAR [113]. This contamination resulted in the binding of the CD19-redirected CAR to the CD19 antigen; therefore, the target antigen was no longer recognizable by the administered CD19-redirected CAR-Ts [113]. Even though this incidence is rare in the case of B-cell malignancies, it has a higher occurrence possibility when generating autologous CAR-Ts from patients with T-cell malignancies (especially in T-cell leukemia patients whose number of circulating malignant T cells are high) [114–117]. In such cases, generating allogeneic CAR-Ts from a healthy third-party donor could be considered a suitable solution. However, allogeneic CAR-Ts also have their caveats. For instance, they might mediate life-threatening GvHD or they may be quickly attacked and eliminated by the immune system of the recipients [118]. Both of these hurdles significantly impair the tumoricidal activity of CAR-Ts [111]. In this regard, researchers have developed allogeneic CAR-Ts to address the mentioned limitations using various strategies including gene-editing methods [118]. It is important to state that allogeneic CAR-Ts have shorter in vivo persistence after infusion to patients in comparison with autologous CAR-Ts [118]. This characteristic of allogeneic CAR-Ts can be beneficial in the prevention of T-cell aplasia in the case of CAR-T therapy of T-cell malignancies [118]. As mentioned earlier, researchers have developed off-the-shelf CD7-redirected CAR-Ts by knocking out TRAC in T cells using CRISPR-Cas9 before viral transduction [59]. These CAR-Ts were also resistant to fratricide due to the knock-out of CD7 [59]. As the results of this study indicated, these CAR-Ts demonstrated effective tumoricidal activity in vitro and in vivo without mediating xenogeneic GvHD [59].

Zinc-finger nucleases (ZFN) and TALEN are two other genome-editing tools that have been utilized for improving off-the-shelf CAR-Ts [119]. However, most of these off-the-shelf CAR-Ts have been investigated in other types of hematologic malignancies [119, 120]. For instance, ZFN has been applied for eliminating the expression of α and β chains of the endogenous TCR in allogeneic T cells for the generation of off-the-shelf CD19-redirected CAR-Ts [120]. These CAR-Ts exhibited specificity against their target antigen and they did not show any response to TCR stimulation [120]. In addition, TALEN-mediated α and β knock-out allogeneic CAR-Ts targeting CS1 and CD22 are currently under clinical investigation for the treatment of patients with MM (NCT04142619) and B-ALL (NCT04150497), respectively [119]. Using such approaches can help generate off-the-shelf allogeneic CAR-Ts that might safely be used in clinical settings for the treatment of patients with hematologic malignancies [119, 120].

Another strategy is the use of multivirus-specific T (multiVST) cells as effector cells for CAR expression. Genetically engineering these cells to be deficient in the expression of the CAR target antigen renders them fratricide-resistant. These CAR-Ts might be beneficial in fighting against viral infections in the case of T-cell aplasia in patients with T-cell malignancies under CAR-T therapy. A clinical study (NCT01570283) has demonstrated that multiVST cells are safe and capable of mediating virological and clinical responses in immunocompromised recipients of allogeneic transplants [121]. Another study has also highlighted the efficacy of multiVST cells when used as prophylaxis in conferring immunity with a low risk of inducing GvHD in a high proportion of immunocompromised patients [122]. Melenhorst et al. have also indicated that adoptive transfer of allogeneic virus-specific T (VST) cells with HLA alloreactivity is safe and does not mediate GvHD in humans [123]. Such data might indicate that using these T cells may be a suitable approach for generating off-the-shelf CAR-Ts. Moreover, the application of multiVST cells may also be beneficial in the case of T-cell aplasia and life-threatening viral infections in patients with T-cell malignancies under CAR-T therapy.

γδ T cells make up for 1 to 5% of circulating lymphocytes [124, 125]. These cells are the prevalent lymphocytes in the skin, reproductive system, and the intestine [124, 125]. This property of γδ T cells is very beneficial in adoptive cell therapy since αβ T cells can poorly access such places [124, 125]. Moreover, in regards to migration towards hard-to-reach tumor sites, γδ T cells express chemokine receptors interacting with tumor cell-secreted chemokines [126]. As a result, this characteristic helps γδ T cells migrate towards tumor sites more easily [126]. Additionally, Vγ9Vδ2 T cells are a subset of γδ T cells that have the ability to attack tumor cells through the recognition of phosphoantigens, including isopentenyl pyrophosphate (IPP) [127, 128]. Since tumor cells accumulate IPP, they can be recognized by Vγ9Vδ2 T cells [127, 128]. Moreover, γδ T cells have the capability to be expanded to large numbers ex vivo [129, 130]. Since the TCR activation of γδ T cells is not MHC-dependent, these cells are considered incapable of mediating GvHD [129, 130]. Moreover, following activation, γδ T cells are capable of acting as proficient antigen-presenting cells [131]. All of these properties render γδ T cells a suitable platform for CAR expression and allogeneic adoptive cell therapy.

So far, studies have used γδ T cells for generating CAR-Ts redirected against various antigens including GD2 and CD19 [131, 132]. In particular, Rischer et al. developed GD2- and CD19-redirected γδ CAR-Ts using retroviral transduction and reported that these cells demonstrated specific and efficient target cell recognition and eradication and INF-γ secretion in vitro [132]. Moreover, Capsomidis et al. also reported similar results and added that CAR expression does not impinge on the migration ability of γδ CAR-Ts towards tumor cells in culture [131]. These researchers also indicated that Vδ1 and Vδ2 subsets can be expanded and transduced ex vivo enabling adequate cell populations for clinical investigations [131]. In 2017, Harrer et al. generated MCSP-redirected γδ CAR-Ts using mRNA transfection [133]. These researchers reported that the cytotoxicity of these CAR-Ts against melanoma cell lines was similar to that of conventional T cells but their overall cytokine production capability was lower [133]. Such reports pave the way for more studies investigating the potential applicability of γδ CAR-Ts as an allogeneic platform, especially in T-cell malignancies. Moreover, a phase I clinical trial (NCT04107142) has assessed the safety and tolerability of haploidentical/allogeneic NKG2DL-redirected γδ CAR-Ts in patients with R/R solid tumors. Another phase I clinical trial (NCT02656147) has investigated the safety, efficacy, and duration of response of CD19-redirected γδ CAR-Ts in patients with R/R CD19^+^ hematologic malignancies. Even though both of these clinical trials have been completed, no official reports of their outcomes have been published yet.

As described in the previous sections, NK cells have different expression levels or do not express some of the TAAs expressed by malignant and normal T cells in various T-cell malignancies [44, 52]. Therefore, they may be used as reliable effector cells for CAR expression minimizing or eliminating the possibility of fratricide. Moreover, in CAR-T therapy of T-cell malignancies, conventional CAR-Ts have long-term persistence after administration which can lead to T-cell aplasia and life-threatening or hard-to-manage consequences. On the other hand, CAR-expressing NKs are not as long-lasting as conventional CAR-Ts [134]. This characteristics of CAR-NKs might obviate the need for using CAR expression and activity controlling platforms or transient CAR expression approaches for minimizing the possibility of T-cell aplasia [134]. Above all this, NK-based CAR-expressing effector cells can eradicate tumor cells in a specific manner without mediating GvHD since they do not possess TCR [135]. However, there are several limitations restricting broader application of NK cells as a platform for CAR expression. The in vitro expansion process of NK cells and the CAR transgene introduction into them are both challenging as compared to those of T cells [136]. As a solution, researchers have used the NK-92 cell line as an alternative [137, 138]. However, the possible tumorigenicity of NK cell lines, such as NK-92, is also another point of concern. In this regard, NK cell lines used as CAR-expressing effector cells are exposed to radiation before administration to patients [137]. It is important to note that this process guarantees the safety of these cells for clinical application but results in a significant reduction of their cytotoxicity [137]. Therefore, the optimal irradiation dose used for this aim is still a subject of ongoing studies [137].

Moreover, Chen and colleagues are among researchers who have reported mortality of xenograft models during in vivo studies of CD3- and CD5-redirected CAR-NKs [44, 52]. These researchers reported that the xenograft models died minutes after CAR-NK administration mainly because of the stroke from the CAR-NK infusion process or NK-92 cell aggregation [44, 52]. Such data point out the necessity that more preclinical and clinical investigations are required to safely conclude that NK cell lines are safe as an allogeneic source of effector cells for the development of CAR-based treatment modalities and their application in clinical settings.

## Conclusion

CAR-T therapy of T-cell malignancies is one of the most intricate and challenging areas of cancer immunotherapy. The very unique and sometimes hard-to-manage challenges of this field are the foremost reason behind the poor clinical responses as compared to the CAR-T therapy of B-cell malignancies. However, the similarities shared between the caveats of the CAR-T therapy of T-cell and B-cell malignancies have led to mutual counterstrategies and solutions. For instance, the prolonged CAR-T persistence in patients with B-cell malignancies after infusion leads to B-cell aplasia. B-cell aplasia is easily managed by immunoglobulin replacement therapy [139, 140]. On the contrary, the extended CAR-T persistence and antitumor activity in patients with T-cell neoplasms can result in T-cell aplasia, which is far more complicated and life-threatening compared with B-cell aplasia. To this day, there have not been many available solutions for preventing T-cell aplasia or its serious consequences. One strategy is bridging the CAR-T recipient to an allogeneic HSCT [19]. Moreover, the strategies of equipping CAR-Ts with safety switches may be beneficial in controlling the expression and antitumor activity of CAR-Ts after administration and preventing persistence-associated adverse events such as T-cell aplasia. These strategies may also be advantageous in many fields of CAR-T therapy other than T-cell malignancies. Moreover, transient approaches for CAR expression have a wide range of applicability in CAR-T therapies. However, all of these strategies require in-depth assessments, particularly in the field of T-cell neoplasms.

As discussed here, genome editing of T cells and generating CAR-Ts that are deficient in the expression of the CAR target antigen may help prevent fratricide. Using alternative effector cells such as NKs may also appear beneficial in this case if the targeted antigen is not expressed by these alternative effector cells. Moreover, the presence of leukemic blasts in the population of T cells after leukapheresis in patients with T-cell malignancies is one of the most challenging hurdles of this field of CAR-T therapy that restricts the use of autologous T cells for CAR-T generation. Using other types of effector cells or using universal off-the-shelf T cells which have been genetically engineered may come as promising solutions. There are numerous tactics for generating off-the-shelf universal CAR-Ts. Some of these approaches have been evaluated in clinical studies and have proven effective as others are still under in-depth assessments [141]. However, allogeneic CAR-Ts have demonstrated several advantages over autologous CAR-Ts [142]. The production of autologous CAR-Ts is challenging, expensive, and time-consuming and the quality of the final product is generally impacted since the patients are critically ill and are under various types of therapy including chemotherapy and radiation therapy [142]. Moreover, generating a sufficient number of autologous CAR-Ts in such situations is not always promised [142]. Allogeneic CAR-Ts do not have these limitations and their greatest hurdle is their ability for GvHD mediation, which has also been managed by genetically disrupting the expression of their TCR or human leukocyte antigen (HLA) [142]. Moreover, using VST or γδ T cells or NK cells lines have been proposed as allogeneic platforms for CAR expression. However, more preclinical and clinical data is required for validating the safety and efficacy of these options. Ultimately, there are still efforts that need to be made to combine suitable counterstrategies for having a CAR-T therapy capable of tackling the mentioned challenges in a particular type of T-cell malignancy.

## Data Availability

Not applicable.

## Abbreviations

CAR-T: Chimeric antigen receptor T-cell
R/R: Relapsed/refractory
FDA: Food and Drug Administration
DLBCL: Diffuse large B-cell lymphoma
B-ALL: B-cell acute lymphoblastic leukemia
FL: Follicular lymphoma
MCL: Mantle cell lymphoma
BCMA: B-cell maturation antigen
MM: Multiple myeloma
T-ALL: T-cell acute lymphoblastic leukemia
LGL: T-cell large granular lymphocyte
ATL or ATLL: Adult T-cell leukemia/lymphoma
T-PLL: T-cell prolymphocytic leukemia
PTCLs: Peripheral T-cell lymphomas
PBMC: Peripheral blood mononuclear cells
NK: Natural killer
TSAs: Tumor-specific antigens
TAAs: Tumor-associated antigens
scFv: Single-chain variable fragment
mAb: Monoclonal antibody
TCR: T-cell receptor
MHC: Major histocompatibility complex
CTCL: Cutaneous T-cell lymphoma
CR: Complete remission
TALEN: Transcription activator-like effector nuclease
PEBL: Protein expression blocker
PDX: Patient-derived xenografts
TRAC: TCR alpha chain
GvHD: Graft-versus-host disease
T-LBL: T-cell lymphoblastic lymphoma
MRD: Minimal residual disease
CRS: Cytokine release syndrome
ALCL: Anaplastic large cell lymphoma
ADC: Antibody–drug conjugate
MMAE: Monomethyl auristatin E
HL: Hodgkin lymphoma
cHL: Classical Hodgkin lymphoma
CCR4: C–C chemokine receptor type 4
Tregs: Regulatory T cells
TRBC1: T-cell receptor beta constant 1
TRBC2: T-cell receptor beta constant 2
NSG: NOD-SCID gamma
T-NHL: T-cell non-Hodgkin lymphoma
AITL: Angioimmunoblastic T-cell lymphoma
HSCT: Hematopoietic stem cell transplantation
HSV-TK: Herpes simplex virus thymidine kinase
ZFN: Zinc-finger nucleases
multiVST: Multivirus-specific T
VST: Virus-specific T
IPP: Isopentenyl pyrophosphate
HLA: Human leukocyte antigen
